# Regulation of mTORC2 Signaling

**DOI:** 10.3390/genes11091045

**Published:** 2020-09-04

**Authors:** Wenxiang Fu, Michael N. Hall

**Affiliations:** 1 Center for Life Sciences, School of Life Sciences, Yunnan University, Kunming 650500, China; 2 Biozentrum, University of Basel, CH4056 Basel, Switzerland; m.hall@unibas.ch

**Keywords:** mTOR, mTORC2 signaling, Akt, signaling crosstalk

## Abstract

Mammalian target of rapamycin (mTOR), a serine/threonine protein kinase and a master regulator of cell growth and metabolism, forms two structurally and functionally distinct complexes, mTOR complex 1 (mTORC1) and mTORC2. While mTORC1 signaling is well characterized, mTORC2 is relatively poorly understood. mTORC2 appears to exist in functionally distinct pools, but few mTORC2 effectors/substrates have been identified. Here, we review recent advances in our understanding of mTORC2 signaling, with particular emphasis on factors that control mTORC2 activity.

## 1. Introduction

The target of rapamycin (TOR) was discovered in yeast nearly 30 years ago [1], and our understanding of TOR signaling has since greatly expanded and keeps growing. Like yeast TOR, mammalian TOR (mTOR, also known as mechanistic TOR [2]) is a central controller of cell growth and metabolism [3,4]. mTOR signaling is physiologically important and its dysregulation is linked to many diseases. It exists in the two structurally and functionally distinct complexes mTORC1 and mTORC2. Compared to mTORC1, much is still unknown about mTORC2. mTORC2 was first described as a rapamycin-insensitive complex that regulates organization of the actin cytoskeleton [5,6], as previously shown for yeast TORC2 [7,8]. Here we review the structure, effectors, localization, and regulation of mTORC2, and discuss future perspectives.

## 2. mTORC2 Composition and Structure

**Figure 1:**
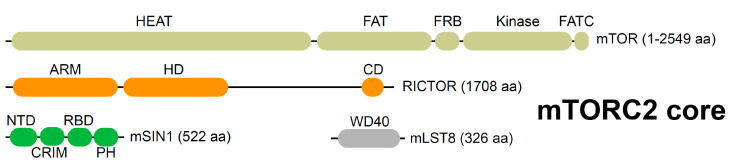
Composition of mTORC2 core. mTORC2 is composed of four core subunits (mTOR, RICTOR, mSIN1, and mLST8). mTOR is composed of several domains, including the HEAT (huntingtin, elongation factor 3, a subunit of protein phosphatase 2A, TOR1; a tandemly repeated motif with helical structure), FAT (FRAP, ATM, TRRAP; α helices arranged as repeats), FRB (FKBP12–rapamycin binding), kinase, and FATC (FRAP, ATM, TRRAP, C-terminal; an α-helix and a disulfide-bonded loop) domains. RICTOR has armadillo (ARM; two curved layers of α-helix) repeats, a HEAT-like domain (HD), and a C-terminal domain (CD), and all three regions likely interact with mSIN1. A flexible region between HD and CD contains most of the identified RICTOR phosphorylation sites. mSIN1 is composed of NTD (N-terminal domain), CRIM (conserved region in the middle; substrate recruitment), RBD (Ras binding domain), and PH (pleckstrin homology; has membrane targeting capacity) domains. NTD and CRIM regions of mSIN1 have direct contact with RICTOR and mLST8. mLST8 is composed mainly of WD40 repeats and interacts with the kinase domain of mTOR.

mTORC1 and mTORC2 share two essential core subunits, the kinase catalytic subunit mTOR and mammalian lethal with SEC13 protein 8 (mLST8). The specific core subunit of mTORC1 is regulatory-associated protein of mTOR (RAPTOR), whereas rapamycin-insensitive companion of mTOR (RICTOR) and mammalian stress-activated Map kinase-interacting 1 (mSIN1) are unique core subunits of mTORC2 (Figure 1). RICTOR mainly has a scaffolding role, and mSIN1 likely contains the substrate binding site and determines subcellular localization of mTORC2. All four core subunits of mTORC2 are essential for mTORC2 activity, but knockout of mLST8 appears not to affect mTORC1 activity [9]. mLST8 loss weakens mTOR interaction with RICTOR and mSIN1 in mTORC2, but not with RAPTOR in mTORC1 [9,10,11]. K63-type polyubiquitination of mLST8 at K305 and K313 prevents mTORC2 assembly and signaling [10]. The mTORC2 structure suggests that mLST8 might help mSIN1 in substrate recruitment and mLST8 ubiquitination could potentially disrupt this [12].

DEP-domain-containing mTOR-interacting protein (DEPTOR) is a regulatory component of both mTORC1 and mTORC2. DEPTOR inhibits mTORC1 and mTORC2, likely via binding of its PDZ domain to mTOR [13]. Proteins associated with RICTOR 1 (PROTOR1) and 2 (PROTOR2) are additional components of mTORC2 [14,15,16], but do not seem to affect intrinsic mTORC2 activity [17]. The Tti1-Tel2 complex, which interacts with mTOR directly, is required for stability of mTOR and assembly of both mTORC1 and mTORC2 [18,19].

The structure of the mTORC2 core has been resolved at intermediate resolution by two independent groups [20,21]. Recently, the 3.2 Å resolution structure of mTORC2 core was defined, with significantly improved building of RICTOR and mSIN1 regions [12]. mTORC2 is a homodimer of mTOR, mLST8, RICTOR, and mSIN1 heterotetramers. mTOR-mLST8 in mTORC2 adopts a similar conformation as in mTORC1. RICTOR interacts and forms multiple contacts with mTOR. The C-terminal domain of RICTOR masks the FKBP-rapamycin binding domain in mTOR, thus explaining the rapamycin insensitivity of mTORC2. RICTOR has an ATP-binding site, but its role is uncharacterized. The N terminus of mSIN1 is an integral component of mTORC2 that connects RICTOR to mLST8. The middle and C-terminal parts of mSIN1, as well as a region of RICTOR (aa 1008–1559) that is predicted to be disordered, remain to be resolved due to high flexibility.

## 3. mTORC2 Effectors

Although many mTORC1 substrates have been identified, mTORC2 is known to phosphorylate mainly AGC kinases [22], including Akt (also known as protein kinase B, PKB), protein kinase C (PKC) family members, and serum- and glucocorticoid-induced kinases 1 (SGK1) at their hydrophobic motif (HM) and turn motif (TM).

### 3.1. AGC Kinase HM

The best characterized kinase activity of mTORC2 is its phosphorylation of Ser473 in the HM of Akt [23,24]. The functional output of Ser473 phosphorylation is context-dependent. For example, in mSIN1 knockout mouse embryonic fibroblasts (MEFs), Akt cannot phosphorylate forkhead box O1/3a (FoxO1/3a), but it can phosphorylate other substrates such as tuberous sclerosis complex 2 (TSC2) and glycogen synthase kinase 3 (GSK-3) [25]. Liver-specific RICTOR knockout disrupts insulin-Akt signaling to FoxO1 and GSK3α/β [26], whereas the phosphorylation of multiple Akt substrates, including FoxO1, GSK3β, AS160, and PRAS40, is not affected by adipose-specific RICTOR knockout [27]. mTOR kinase inhibitors PP242 and Torin1 were able to reduce the phosphorylation of PRAS40 but not GSK3β in rat adipocytes [28]. Inhibition of mTORC2 has no effect on phosphorylation of GSK3β at Ser9 in many contexts because GSK3β can be phosphorylated by kinases other than Akt, such as S6K, PKC, and Aurora A [29,30,31]. Chronic disruption of mTORC2 may cause compensatory changes, and specificity of chemical inhibitors is also a concern. Nevertheless, these findings suggest that mTORC2 phosphorylation of Ser473 is required for context dependent Akt activity toward some, but not all, substrates. Indeed, the direct contribution of Ser473 phosphorylation to Akt activity was recently assessed. It has been proposed that Ser473 phosphorylation activates Akt by disrupting an autoinhibition conformation [32]. A recent study in adipocytes showed that mutation of Akt2 Ser474 (homologous to Ser473 in Akt1) reduced Akt2 activity by about 50% against its substrates TSC2, PRAS40, FoxO1/3a, and AS160 [33]. This suggests a role of Ser473 phosphorylation in promoting maximal Akt activity, rather than determining substrate specificity. Diffusion measurements in live cells showed that phosphorylated, active Akt is restricted mainly to cellular membranes [34]. The exact effects of mTORC2 on endogenous Akt toward its substrates in different contexts inside cells need further study.

Besides Akt, mTORC2 also phosphorylates the HM of PKCs and SGK1. The PKC family can be divided into three groups: the conventional c-PKCs (α, β and γ), the novel n-PKCs (δ, ε, η, θ, and μ), and the atypical a-PKCs (ι and ζ). mTORC2 selectively mediates phosphorylation of the HM of c- and *n*-PKCs, including types α, β, γ, and ε [35,36]. PKCα HM phosphorylation by mTORC2 regulates actin polymerization [5,6]. In addition, mTORC2 phosphorylates the HM in SGK1 and thereby activates SGK1 [37,38].

### 3.2. AGC Kinase TM

mTORC2 associates with the ribosome to phosphorylate nascent Akt at its TM (Thr450) during translation [39]. This phosphorylation event prevents premature Akt ubiquitination and increases the stability of nascent Akt polypeptide. As is the case with the HM, TM phosphorylation of c-PKCs (α, β, and γ) and n-PKC (ε) is mediated by mTORC2 [35,36]. mTORC2 also phosphorylates the TM of an atypical PKC, type ζ [40]. TM phosphorylation of PKCα is required for its maturation and stability. Whether mTORC2 phosphorylates the TM in SGK1 is not clear.

### 3.3. Other mTORC2 Effectors

Only a limited number of mTORC2 substrates have been identified. Besides classical HM and TM sites, mTORC2 also phosphorylates Ser477 and Thr479 at the C terminus of Akt under growth stimulation conditions. These two sites are proposed to be essential for Akt activity, and can be alternatively phosphorylated by Cdk2/Cyclin A during cell cycle progression [41,42]. The ubiquitin ligase subunit F-box and WD repeat domain-containing 8 (FBW8) mediates the degradation of insulin receptor substrate-1 (IRS-1), and mTORC2 stabilizes FBW8 by phosphorylating it at Ser86. mTORC2 thus negatively feeds back to IRS-1 to diminish insulin signaling [43]. mTORC2 promotes insulin-like growth factor 2 (IGF2) production by co-translationally phosphorylating IGF2 mRNA-binding protein 1 (IMP1) at Ser181 [44]. Although mTORC2 has been proposed to have tyrosine kinase activity against insulin receptor (InsR) and insulin-like growth factor 1 receptor (IGF1R), the involved mechanisms need to be further clarified [45].

Like mTORC1, mTORC2 is also a central hub for cell metabolism involving amino acids, glucose, nucleotides, fatty acids, and lipids [46,47,48]. The cysteine–glutamate antiporter xCT, also known as solute carrier family 7 member 11 (SLC7A11), is a 12-pass transmembrane protein that takes up cysteine in exchange for glutamate. mTORC2 can inhibit the activity of xCT via phosphorylation of xCT Ser26 [49]. Defining mTORC2 effectors has relied largely on genetic studies, and many roles of mTORC2 in metabolism are likely attributable to indirect downstream effectors. For example, mTORC2 promotes ATP-citrate lyase (ACLY) phosphorylation at Ser455, but this is likely mediated via Akt HM phosphorylation. ACLY phosphorylation increases carbohydrate response element binding protein (ChREBP) activity, histone acetylation, and gluco-lipogenic gene expression in brown adipocytes [50,51]. mTORC2 also regulates chemokine CXCL12-mediated angiogenesis via Akt HM phosphorylation [52]. Another mTORC2 effector, NDRG1 (*N*-Myc downstream regulated 1), is phosphorylated by the mTORC2 substrate SGK1 [37]. The cellular effect of this phosphorylation is unclear; however, NDRG1 is generally upregulated by stress signals, and could promote or suppress tumor formation under certain conditions [53].

mTORC2 is required for other metabolic events. mTORC2 can regulate glycolysis and the pentose phosphate pathway (PPP) via Akt and c-Myc. Akt phosphorylation by mTORC2 promotes glucose uptake [54] and hexokinase 2 mediated glucose conversion to glucose-6-phosphate [55], the initial steps of glycolysis. Glucose-6-phosphate can be alternatively used by PPP for ribose-5-phosphate and nicotinamide adenine dinucleotide phosphate (NADPH) production. mTORC2 upregulates c-myc through FoxO acetylation, and controls the expression of multiple enzymes in glycolysis and PPP [56]. mTORC2 is required for acetylation of FoxOs, and this is likely mediated via Sirtuin6 suppression or inhibitory phosphorylation of class IIa histone deacetylases [56,57].

As noted above, direct and indirect effectors of mTORC2 contribute to complex cellular functions, involving growth factor signaling and cell metabolism. Although mTORC2 plays important roles in physiology and in pathophysiology such as metabolic diseases and cancer [58,59,60,61], in many cases we still await a complete understanding of the underlying direct molecular links.

## 4. Subcellular Localization of mTORC2

Distinct pools of mTORC2 at different subcellular locations could underlie mTORC2 signaling through different effectors [62]. Accumulating evidence supports the notion that mTORC2 localizes to the cytoplasm, nucleus, plasma membrane, endosome, Golgi body, ribosome, lysosome, endoplasmic reticulum (ER), mitochondria, and mitochondria-associated ER membrane (MAM) [63,64]. A so-called LocaTOR2 reporter was recently developed to detect mTORC2 kinase activity toward Akt Ser473 at different locations [65]. FRB-Akt2 is induced to be recruited by different FKBP-mCherry fusions to six different membrane compartments, and the phosphorylation of FRB-Akt2 Ser473 is used as the readout for the localized mTORC2. Using this reporter, mTORC2 was found to be active mainly at the plasma membrane, mitochondria, and a subpopulation of endosomal vesicles. The observation of mTORC2 activity in mitochondria supports the many reported phenotypic connections between mTORC2 and mitochondria [66,67,68]. mTORC2 localizes to MAM upon stimulation by growth factors [55]. Another report indicated that the association of mTORC2 with the ribosome is also growth factor-dependent [69]. Since ribosomes exist at the ER, and MAM is a connection site for mitochondria and ER, it is reasonable to hypothesize that the detected mitochondrial activity of mTORC2 could be linked to its localization to MAM and ribosomes.

Different isoforms of mSIN1 have diverse locations and constitute at least three distinct mTORC2 pools [70,71]. At least two mSIN1 isoforms have the pleckstrin homology (PH) domain with phosphoinositide-binding activity. Although there is controversy regarding which phosphoinositide species bind to the PH domain, the PH domain is important for the localization of mSIN1, and thereby mTORC2, to the plasma membrane [65,72,73]. The mSIN1 isoform, with a truncated C terminus, does not have a PH domain but still forms a complex with other mTORC2 subunits. Not surprisingly, this truncated isoform does not localize to the plasma membrane. Only mTORC2 with a PH domain responds to insulin, so it is reasonable to suggest that the PH domain of mSIN1 determines the localization of mTORC2 and is important for mTORC2 activation by insulin. Whether and how the localization of mTORC2 to the plasma membrane is regulated in response to different cues remains to be determined.

While mTORC2 activity toward other substrates/sites exists, the full spectrum of mTORC2 localization is likely underestimated using the LocaTOR2 reporter. Apart from the need to assess mTORC2 activity at other subcellular locations, mTORC2 activity against certain substrates, such as Akt TM phosphorylation, cannot be reflected by this reporter. Assembly of mTORC2 could be dynamic and mTORC2 could potentially translocate from one site to another in response to different cues.

## 5. TORC2 Regulation by Plasma Membrane Tension in Yeast

TORC2 is conserved in eukaryotes. The core subunits of TORC2 in the budding yeast *Saccharomyces cerevisiae* are Tor2, Lst8, Avo3, and Avo1, with mTOR, mLST8, RICTOR, and mSIN1 as their mammalian orthologues. Both mTORC2 and yeast TORC2 assemble into rhomboid-shaped dimers. Avo1 is 1176 aa, whereas the longest isoform of its mammalian counterpart mSIN1 is only 522 aa. They both have homologous PH domains that bind membranes, but mSIN1 has a much shorter N-terminal region that connects with RICTOR and mLST8 in mTORC2. This might contribute to the functional differences between mammalian and yeast TORC2.

Unlike mTORC2, which is found at many different subcellular locations, TORC2 in yeast localizes mainly to specific regions of the plasma membrane, termed membrane compartments containing TORC2 (MCTs) [74]. TORC2 activity is regulated by plasma membrane tension. Plasma membrane stress, induced by either mechanical stretching or inhibition of lipid synthesis, leads to redistribution of Slm proteins from invaginations called eisosomes to MCTs. Slm proteins then promote TORC2-mediated Ypk1 phosphorylation, likely via recruitment of Ypk1 to the plasma membrane [75,76]. Interestingly, decreased plasma membrane tension triggers phase separation of phosphatidylinositol-4,5-bisphosphate (PI(4,5)P_2_) into invaginated membrane domains, which cluster and inactivate TORC2 [77]. Together, these observations suggest that increased and decreased membrane tension are sensed by mTORC2 via distinct mechanisms.

## 6. Regulation Cues for mTORC2 Activity

**Figure 2:**
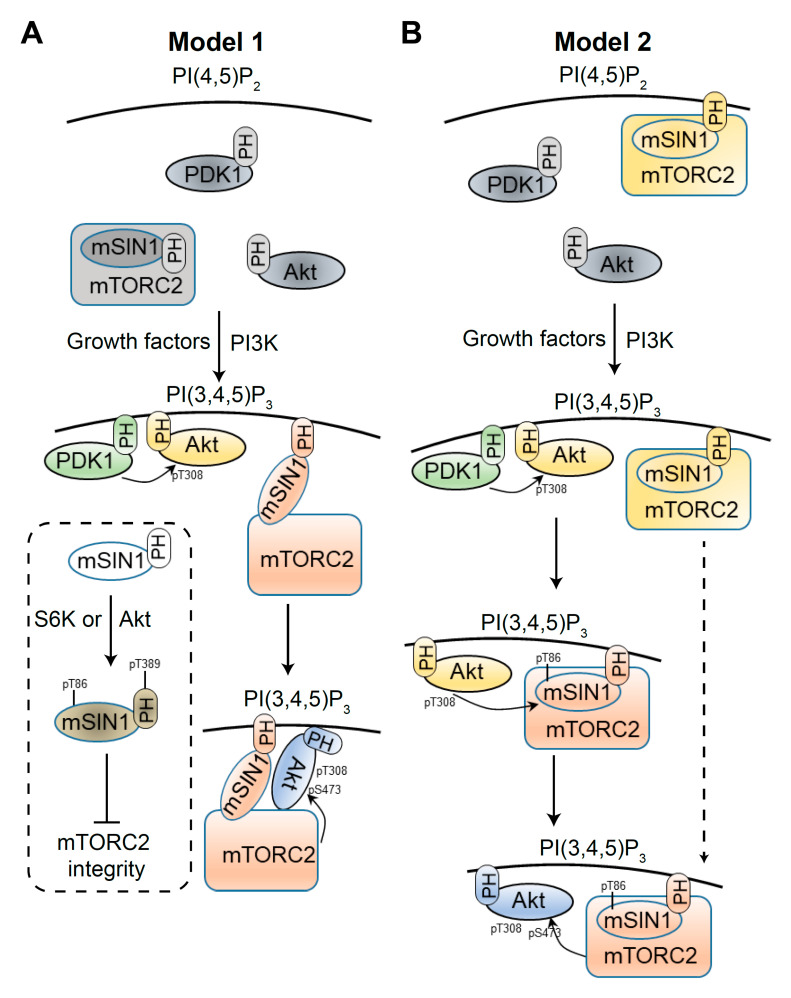
Growth factors induce Akt Ser473 phosphorylation via mTORC2. In both models, in the absence of growth factors, PDK1 and Akt are localized at the cytoplasm. Upon growth factor stimulation, PI(4,5)P_2_ is converted to PI(3,4,5)P_3_ via PI3K at the plasma membrane, and PDK1 and Akt are recruited to the plasma membrane via their pleckstrin homology (PH) domains, leading to phosphorylation of Akt Thr308 by PDK1. In model 1 (**A**), PI(3,4,5)P_3_ recruits mTORC2 to the plasma membrane and directly activates mTORC2 toward Akt Ser473 via the release of an inhibitory conformation. In the box, S6K or Akt leads to dual phosphorylation of mSIN1 at Thr86 and Thr389 to inhibit mTORC2. In model 2 (**B**), mTORC2 permanently resides at the plasma membrane. Akt-pThr308 phosphorylates mSIN1 Thr86 within mTORC2, which in turn boosts mTORC2 activity. mTORC2 then phosphorylates Akt Ser473, leading to full activation of Akt. The contribution of mTORC2 containing Thr86-nonphosphorylated mSIN1 to Akt Ser473 phosphorylation is not very clear.

Compared with mTORC1, the regulation of mTORC2 activity is far less understood. Nevertheless, it is clear that the phosphoinositide 3-kinase (PI3K) pathway mediates growth factor-induced Akt Ser473 phosphorylation by mTORC2 (Figure 2). Recent evidence suggests that mTORC2 is also activated by several other cues.

### 6.1. Growth Factor-Mediated Akt Phosphorylation at the Plasma Membrane

Numerous studies report that PI3K inhibition abolishes Akt phosphorylation at both Thr308 and Ser473, the latter of which is an mTORC2 target site. Mechanistically, upon growth factor stimulation, PI3K phosphorylates PI(4,5)P_2_ at the plasma membrane to produce phosphatidylinositol 3,4,5-trisphosphate (PI(3,4,5)P_3_), which is counteracted by phosphatase and tension homolog (PTEN). Akt and its activating kinase PDK1 specifically bind to PI(3,4,5)P_3_ via their PH domains, and are recruited to the plasma membrane, leading to phosphorylation of Akt by PDK1 at Thr308. However, the mechanism by which mTORC2 phosphorylates Akt Ser473 is controversial.

It has been proposed that PI(3,4,5)P_3_ directly activates mTORC2 [72]. As shown in model 1 (Figure 2A), the mSIN1 PH domain binds the mTOR kinase domain and auto-inhibits mTORC2. PI(3,4,5)P_3_, but not the other PIPs, binds the mSIN1 PH domain and recruits mTORC2 to the plasma membrane. In addition, the binding of PI(3,4,5)P_3_ to the mSIN1 PH domain releases the inhibition of mTORC2 by mSIN1, in agreement with a previous study showing that PI(3,4,5)P_3_ directly promotes mTORC2 activity *in vitro* [78]. With Akt Thr308 being phosphorylated by PDK1, the plasma membrane-localized and activated mTORC2 further phosphorylates Akt at Ser473.

Two key ideas of the above model are as follows. First, the localization of mSIN1, and thereby mTORC2, to the plasma membrane is PI(3,4,5)P_3_-dependent. Second, PI(3,4,5)P_3_ induces a structural change in mTORC2 that increases its activity. However, a recent study using the LocaTOR2 reporter challenged this model and suggested that mTORC2 permanently resides at the plasma membrane and is constitutively active [65]. Unlike Akt, the localization of mSIN1 was shown not to change with growth factor stimulation or PI3K inhibition. Slightly contrary to model 1, a previous study showed that the mSIN1 PH domain binds many different PIPs, including PI(4,5)P_2_ and PI(3,4,5)P3 [73]. Once exogenous Akt2 is recruited to the plasma membrane, the LocaTOR2 reporter indicated that mTORC2 activity is not affected by growth factors or PI3K. In agreement with this, myristoylated Akt localizes to membranes and is constitutively active with phosphorylated Ser473 [79,80]. Thus, translocation of Akt to the plasma membrane is both necessary and sufficient for its phosphorylation by mTORC2.

Growth factor stimulated PI(3,4,5)P_3_ could indirectly promote mTORC2 activity. Phosphorylation of mTOR Ser2481 might be involved, but its role is poorly characterized [81,82]. It was shown that Akt phosphorylates mSIN1 Thr86 to activate mTORC2 in 3T3-L1 adipocytes [83]. The same group later confirmed Akt as the major kinase for mSIN1 Thr86 phosphorylation in HeLa cells and in several other contexts [84]. This positive feedback loop between Akt and mTORC2 supports another layer of mTORC2 activation induced by growth factors. Combined with the LocaTOR2 study, model 2 is proposed (Figure 2B), in which Akt Ser473 phosphorylation is mediated by both translocation of Akt to the plasma membrane and an increase of mTORC2 activity by Akt. However, whether mSIN1 Thr86 phosphorylation is a prerequisite for mTORC2 activity is not clear. If not, how much mTORC2 containing Thr86-nonphosphorylated mSIN1 contributes to total Akt Ser473 phosphorylation inside cells is unknown.

The regulation of mTORC2 activity by mSIN1 phosphorylation is controversial. In another study [85], the major kinase for mSIN1 Thr86 phosphorylation was confirmed to be Akt in 3T3-L1 adipocytes [83] and S6K in HeLa cells. This study also found that phosphorylation of another mSIN1 site, Thr389, requires both Akt and S6K in 3T3-L1, although it is mainly dependent on S6K in HeLa cells. Phosphorylation of mSIN1 on both Thr86 and Thr398 further disassembles mTORC2 and suppresses mTORC2 activity (Figure 2A), suggesting an mTORC1-to-mTORC2 negative feedback loop operating via S6K. The discrepancies in the major kinase for mSIN1 phosphorylation and the effects of mSIN1 phosphorylation on mTORC2 activity need further study.

### 6.2. mTORC2 Activity Is Regulated by Its Subcelluar Localization

#### 6.2.1. mTORC2 Is Active Mainly at Membrane Compartments

**Figure 3:**
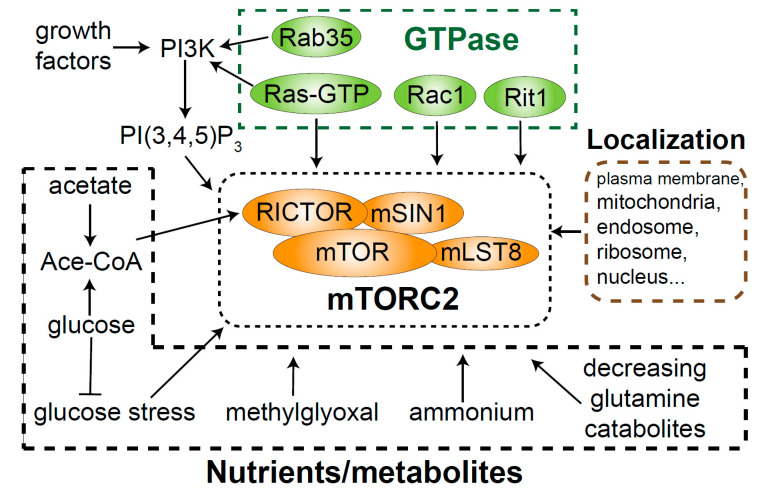
Upstream of mTORC2 signaling. Besides canonical PI3K-mediated mTORC2 activation, mTORC2 activity is controlled by its localization to different subcellular compartments, small GTPases, nutrients, and metabolites.

mTORC2 exists at multiple subcellular locations, but how mTORC2 subpopulations are differentially regulated is poorly characterized (Figure 3). The LocaTOR2 reporter indicates that mTORC2 is active mainly at the plasma membrane, outer mitochondrial membrane, and endosomal vesicles [65]. mTORC2 activity cannot be detected in the cytoplasm or endoplasmic reticulum (ER), and this is likely because either mTORC2 is not active or the counteracting phosphatases limit mTORC2 activity at these locations. Sustained activation of the mTORC2 effector Akt requires binding of PI(3,4,5)P_3_ or PI(3,4)P_2_ to the Akt PH domain, which protects membrane-localized Akt from dephosphorylation [34]. mTORC2 at the plasma membrane is discussed in Section 6.1.

#### 6.2.2. Mitochondria, ER, and MAM 

There are many lines of evidence linking mTORC2 to mitochondria and the ER (reviewed in [63]). In addition, upon growth factor stimulation, mTORC2 localizes to MAM and phosphorylates Akt, which in turn phosphorylates MAM-associated proteins, including the inositol 1,4,5-trisphosphate receptor and hexokinase 2 [55]. MAM is a specialized region of the ER that abuts mitochondria, so the mTORC2 subpopulation at MAM is associated with both mitochondria and the ER. However, these three mTORC2 subpopulations seem to be different. mTORC2 at MAM is growth factor sensitive, but the LocaTOR2 reporter indicates that mTORC2 is active at mitochondria but not ER, and that mitochondria-localized mTORC2 is insensitive to PI3K and growth factors [65].

#### 6.2.3. Ribosome

It was first noticed that mTORC2 associates with actively translating ribosomes [39] where it phosphorylates the TM (Thr450) in nascent Akt. Another study showed that active mTORC2 is associated with the ribosome, and the ribosome is required for mTORC2 activation [69]. The mTORC2–ribosome association is induced by insulin-stimulated PI3K signaling and is independent of protein synthesis. Although ribosome-bound mTORC2 might exist at MAM, the underlying mechanism by which ribosome association activates mTORC2 is unknown.

#### 6.2.4. Endosome and Lysosome

The LocaTOR2 reporter indicates that mTORC2 is active at early and late endosomes, but not at recycling endosomes [65]. Indeed, it was found that the endosomal protein Appl1 is required for Akt HM phosphorylation in zebrafish [86]. Nevertheless, it is unclear whether zebrafish TORC2 is directly activated on endosomes in this context.

The discovery of the lysosome as a center for mTORC1 signaling has greatly improved our understanding of how mTORC1 senses external nutrients and growth factors. Unexpectedly, it was shown that perinuclear clustering of lysosomes delays reactivation not only of mTORC1, but also of mTORC2 and Akt upon serum replenishment [87]. Another study showed that lysosomal mTORC2 activates Akt to inhibit chaperone-mediated autophagy [88]. These findings further suggest that different pools of mTORC2 exist and are regulated differently.

#### 6.2.5. Nucleus

mTORC2 has been suggested to translocate between the nucleus and cytoplasm [89,90]. Inhibition of c-PKC catalytic activity disrupts the localization of mSIN1 and SGK1 to the nucleus and perinuclear compartment, and this further prevents SGK1 Ser422 phosphorylation by mTORC2 [62]. Although the nuclear function of mTORC2 is still not clear, the subcellular distribution of mTORC2 to the nucleus and perinuclear compartment may be relevant to its downstream effector SGK1.

### 6.3. GTPase Activation of mTORC2

Recent studies suggest the involvement of small GTPases in mTORC2 activation (Figure 3). GTPases can activate mTORC2 indirectly via PI3K. Ras-GTP, but not Ras-GDP, interacts with PI3K and increases its activity [91]. Rab35, another small GTPase, was identified in an shRNA screen as a regulator of growth factor-stimulated PI3K signaling [92], which then activates mTORC2.

Direct roles of GTPases in mTORC2 activation have been recognized only recently. Rac1 is a member of the Rho family of GTPases and binds directly to mTOR. In response to growth factor stimulation, Rac1 was shown to regulate the activity of both mTORC1 and mTORC2, surprisingly, independently of its binding to GTP or GDP [93]. Oncogenic Ras with impaired GTPase activity leads to constitutively active Ras signaling. Ras-GTP directly binds mSIN1 and mTOR in mTORC2, which in turn leads to mTORC2 activation at the plasma membrane [94]. The discovery of mTORC2 as a direct Ras effector suggests a key module for tumorigenesis driven by Ras mutation. However, whether direct mTORC2 activation by Ras-GTP is downstream of growth factors or PI3K is not clear. Another member of the Ras family of GTPases, Rit, interacts with mSIN1 and mediates mTORC2 activation following oxidative stress [95]. 

Interestingly, comparable and detailed roles of Ras-GTP in TORC2 activation in *Dictyostelium* were found. The small GTPase Rap1 binds TORC2 component RIP3/SIN1 and regulates RasC-mediated activation of TORC2 [96]. In another study, in response to G protein-coupled receptor activation by chemoattractant, GDP-bound RacE is phosphorylated by GSK-3 and forms a super complex with RasC-GTP and TORC2 to promote Akt phosphorylation [97]. Compared with *Dictyostelium*, the contribution of Ras to TORC2 activation in mammals seems to be minor [94]. This is possibly due to the complex subcellular distribution of mTORC2.

### 6.4. Nutrients and Metabolites

Although there is evidence showing that amino acids activate mTORC2 [98], it is widely accepted that mTORC1 is sensitive to both nutrients and growth factors, whereas mTORC2 is mainly regulated by growth factors. However, recent studies support a role of mTORC2 in metabolic reprograming and show how mTORC2 responds to nutrients (Figure 3).

A decrease in glutamine catabolites due to nutrient deprivation can activate mTORC2 to increase the expression of glutamine–fructose-6-phosphate amidotransferase 1 (GFAT1). mTORC2 thus modulates a glutamine-requiring biosynthetic pathway to mediate metabolic homeostasis [99]. Acetyl coenzyme A (Acetyl-CoA) is the acetyl group donor in protein acetylation, and RICTOR is acetylated at multiple sites. p300-mediated acetylation of RICTOR increases mTORC2 activity, whereas deacetylases inhibit RICTOR acetylation and mTORC2 activity [100]. Glucose and acetate can both replenish acetyl-CoA and activate mTORC2 via RICTOR acetylation [101]. In contrast, glucose stress also activates mTORC2, and its relevance to acetylation is unclear [102]. One possible explanation is that glucose starvation activates 5′ adenosine monophosphate-activated protein kinase (AMPK), which increases mTORC2 activity (see below). Methyglyoxal, a metabolite produced mainly by glycolysis, and the metabolic waste product ammonium both activate mTORC2, but the mechanisms are unclear [103,104]. It is worth mentioning that most of these metabolic inputs are in a stress-like state, so mTORC2 could potentially mediate cell survival in starvation or even toxic conditions. The endogenous levels of these metabolites in physiological or pathological conditions, and their contributions to mTORC2 activity in specific contexts, should be investigated.

## 7. Signaling Crosstalk Regulates mTORC2 Activity

**Figure 4:**
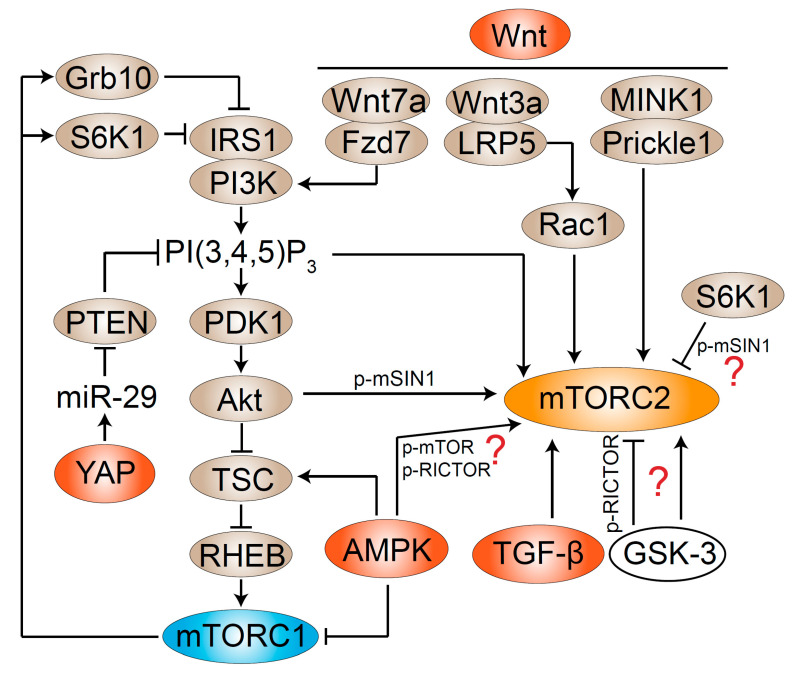
Regulation of mTORC2 activity by signaling crosstalk. AMPK, Wnt, YAP, and TGF-β (depicted in red) positively regulate mTORC2 activity. mTORC1 (depicted in blue) negatively regulates mTORC2 activity.

While most studies on mTORC2 investigate its regulation by PI3K, recent studies revealed that mTORC2 activity is fine-tuned via other signaling pathways (Figure 4).

### 7.1. Feedback Control between mTORC1 and mTORC2

Feedback control loops exist between mTORC1 and mTORC2 [105]. mTORC1 negatively regulates mTORC2 via S6K1. S6K1, downstream of mTORC1, leads to inhibitory phosphorylation of IRS1 and also downregulation of IRS1 protein level [106,107,108]. Downregulation of insulin-PI3K signaling then inactivates mTORC2. S6K1 can also phosphorylate RICTOR at Thr1135, but the functional consequence of this phosphorylation is controversial, and it does not seem to affect mTORC2 kinase activity [109,110,111,112]. S6K1 can phosphorylate mSIN1 at Thr86 and Thr389 to inhibit mTORC2 integrity, but this is controversial (discussed in Section 6.1, Figure 2A).

mTORC1 regulates mTORC2 in another negative feedback loop via growth factor bound-receptor protein 10 (Grb10) [113,114]. mTORC1 phosphorylates Grb10 at multiple sites and stabilizes the protein. Grb10 inhibits phosphorylation of InsR and IRS-1/2, destabilizes IRS-1, and mediates the inhibition of PI3K signaling and mTORC2 activity.

### 7.2. AMPK Activates mTORC2

AMPK, a master regulator of cell energy homeostasis, is activated when there is a lack of energy or nutrients [115]. AMPK downregulates mTORC1 in two ways. First, TSC2 phosphorylation by AMPK likely increases the GTPase-activating protein (GAP) activity of the TSC complex toward the GTPase Ras homolog enriched in brain (RHEB) and thereby inhibits mTORC1 [116]. Second, AMPK directly phosphorylates RAPTOR to inactivate mTORC1 [117]. Given the negative feedback loops between mTORC1 and mTORC2, AMPK-mediated mTORC1 inactivation may lead to mTORC2 activation indirectly (see Section 7.1).

AMPK activates mTORC2 directly in response to energetic stress [118]. AMPK-activating agonists promote mTORC2 signaling independently of mTORC1-mediated negative feedback. AMPK phosphorylates mTOR and RICTOR, and this is sufficient to increase mTORC2 kinase activity. The mechanisms by which this phosphorylation regulates mTORC2 activity, as well as the exact phosphorylation sites, have yet to be fully defined. The direct input of AMPK to mTORC2 suggests a new strategy for cell survival to cope with acute energetic stress.

### 7.3. Wnt Activates mTORC2

The canonical Wnt pathway leads to accumulation of β-catenin in the cytoplasm, followed by its translocation into the nucleus to function as a transcription activator. The noncanonical planar cell polarity (PCP) pathway, which involves Wnt but does not require β-catenin, results in reorganization of the actin cytoskeleton [119]. Wnt3A activates mTORC2 to induce glycolysis during osteoblast differentiation [120]. Low-density lipoprotein receptor-related protein 5 (LRP5), rather than the canonical Wnt signaling component β-catenin, appears to be the principle mediator for this process. Indeed, Wnt3A induces recruitment of Rac1 to the plasma membrane, and Wnt-LRP5 signaling activates mTORC2 via Rac1. In skeletal muscle, Wnt7a binds directly to its receptor Frizzled-7 (Fzd7) to activate the PCP pathway. The association of the Fzd7 receptor complex with PI3K likely leads to the activation of PI3K, and mTORC2 as well [121]. Prickle-like protein 1 (Prickle1) is a PCP protein phosphorylated by the misshapen-like kinase 1 (MINK1). Phosphorylated Prickle1 forms a complex with mTORC2 through binding RICTOR, and further activates mTORC2 to regulate focal adhesion and cancer cell migration [122].

### 7.4. Other Signaling Pathways

Other signaling pathways have also been shown to regulate mTORC2 indirectly or in a context-dependent manner. YAP activates mTORC2. YAP is the main downstream target of the mammalian Hippo pathway in the regulation of tissue growth [123]. It has been shown that YAP directly induces the expression of miR-29 to inhibit translation of PTEN, which antagonizes PI3K [124]. YAP therefore enhances the production of PI(3,4,5)P_3_, which further activates mTORC2 and mTORC1. Thus, YAP connects Hippo and mTOR signaling.

TGF-β activates mTORC2. TGF-β induces epithelial–mesenchymal transition (EMT), a reprograming process widely existing in cancer, involved with cell shape and behavior changes. In cells undergoing EMT, TGF-β induces mTORC2 activity and mTORC2 is required for EMT-associated cell migration and invasion [125]. Pharmacological inhibition of mTOR or knockdown of RICTOR dampens TGF-β-induced differentiation of human myofibroblasts, suggesting that mTORC2 acts downstream of TGF-β signaling [126].

GSK-3 is a substrate of Akt and can regulate mTORC2 activity. Under ER stress, RICTOR phosphorylation at Ser1235 by GSK-3 interferes with mTORC2 binding to its substrate Akt [127]. GSK-3 also directly phosphorylates RICTOR at another site, Thr1695. Phosphorylated RICTOR is then recognized by the E3 ubiquitin ligase F-Box and WD repeat domain-containing 7 (FBW7) and is degraded [128]. Although GSK-3 was reported to suppress mTORC2 activity in both studies, it has been reported to positively affect mTORC1 and mTORC2 in neurons [129]. Whether GSK-3 promotes or inhibits TORC2 in *Dictyostelium* is also controversial [97,130,131,132].

## 8. Perspectives

Recent studies have indicated that mTORC2 has various subcellular locations, including distinct membrane compartments, but the precise localization of mTORC2 is still elusive, and how different pools of mTORC2 coordinate with each other is unclear. Whether and how sub-pools of mTORC2 sense and transduce distinct cues also remain largely unknown. mTORC2 activity is regulated by diverse factors such as growth factors, metabolites, and signaling crosstalk. Molecular details of this complex regulation process still need to be elucidated. Further studies are needed to obtain integrated mechanisms and comprehensive understanding of mTORC2 activation. Finally, it is worth noting that most mTORC2 studies have used Akt Ser473 phosphorylation as the readout of mTORC2 activity, due to the availability of effective antibodies that recognize Akt pSer473. This greatly neglects the fact that mTORC2 phosphorylates other effectors, possibly in response to other conditions [64]. 

Recent ‘omics’ studies have greatly advanced our understanding of mTOR signaling. Proteomics and phosphoproteomics contributed to the identification of novel substrates, interactors, and functions. Emerging evidence supports many undefined links of mTORC2 to membranes. Although analyzing membrane proteins, especially plasma membrane proteins, is always challenging using a mass spectrometry approach, optimized methods and new tools need to be developed [133]. Structural resolution of mTORC2 with membranes, its substrates, or its upstream regulators will further improve our understanding of mTORC2 signaling. Specific small molecules targeting mTORC2 still need to be developed [134]. They will be useful not only to study mTORC2 signaling, but also have therapeutic potential.

Hyperactive mTORC2 signaling is commonly found in cancer, and is tumorigenic in various in vitro and in vivo models [135]. Several upstream regulators and downstream effectors of mTORC2 are well-known tumor suppressors or oncoproteins. Furthermore, RICTOR is overexpressed in adenocarcinoma, glioma, sarcoma, and many other cancer types. RICTOR overexpression promotes assembly of the mTORC2 complex, and its overexpression results from at least two mechanisms, genomic amplification and miRNA regulation (reviewed in [136]). In the future, defining the regulation of mTORC2 signaling may reveal novel drug targets [137].
